# Chinese herbal medicine reduced the risk of stroke in patients with Parkinson’s disease: A population-based retrospective cohort study from Taiwan

**DOI:** 10.1371/journal.pone.0203473

**Published:** 2018-09-07

**Authors:** Ching-Yuan Lai, Jen-Huan Chiang, Jaung-Geng Lin, Hung-Rong Yen, Cheng-Hao Tu, Yi-Hung Chen

**Affiliations:** 1 School of Chinese Medicine, China Medical University, Taichung, Taiwan; 2 Department of Emergency Medicine, China Medical University Hospital, Taichung, Taiwan; 3 Health Data Management Office, China Medical University Hospital, Taichung, Taiwan; 4 School of Medicine, China Medical University, Taichung, Taiwan; 5 Research Center for Traditional Chinese Medicine, Department of Medical Research, China Medical University Hospital, Taichung, Taiwan; 6 Department of Chinese Medicine, China Medical University Hospital, Taichung, Taiwan; 7 Chinese Medicine Research Center, China Medical University, Taichung, Taiwan; 8 Graduate Institute of Acupuncture Science, China Medical University, Taichung, Taiwan; 9 Department of Photonics and Communication Engineering, Asia University, Taichung, Taiwan; Fudan University, CHINA

## Abstract

Parkinson’s disease (PD) is associated with a significantly increased risk of stroke. Traditional Chinese medicine (TCM) has long been used in Asia to treat stroke, but there are no large-scale clinical data to confirm its efficacy in protecting PD patients against stroke. Herein, we analyzed a cohort of 1,000,000 records from Taiwan’s National Health Insurance Research Database for the period 1997–2011, and identified 1,882 patients with new-onset PD. We matched 290 patients who received Chinese herbal medicine (CHM) by age, sex, year of CHM prescription, and year of PD diagnosis with 290 patients who did not use CHM as control. Both cohorts were followed until the end of 2013 for the incidence of new-onset stroke. In a multivariable Cox proportional hazard model adjusted for potential comorbidities, the incidence of stroke was lower among PD patients using CHM compared with non-CHM users (11.10 per 100 person-years vs 23.15 per 100 person-years; Hazard ratio: 0.56; 95% confidence interval:  0.44 to 0.72). The probability curve generated from our follow-up data showed that PD patients receiving CHM treatment had a decreased risk of stroke compared with those not receiving CHM treatment (P <0.001). The analysis on the prescription pattern of CHM revealed that Danshen is the most common single herb and Ma Zi Ren Wan is the most common herbal formula. Although the analysis are limited by a lack of analytic information regarding lifestyle patterns, biochemical profiles, and levels of PD severity in database, this population-based study suggest that CHM may be an complementary therapy to reduce the risk of stroke in PD patients.

## Introduction

Parkinson's disease (PD) is a slowly progressive, irreversible neurodegenerative disease of the central nervous system that affects about 1% of people aged 65 years and over worldwide [1]. Globally, the incidence of PD ranges from 10–18 per 100,000 person-years [1]. Aging populations and rising life expectancies worldwide have led to the prediction that the number of people aged over 50 years with PD in Western Europe’s five most and the world’s 10 most populous nations will double from between 4.1 and 4.6 million in 2005 to between 8.7 and 9.3 million by 2030 [2]. In 2016, 13.20% of the Taiwanese population was aged 65 years and over [3], and Taiwan’s census data reveal average life expectancies of 76.81 years for males and 83.42 years for females [4]. As Taiwan progressively steps into an ‘aged’ country, PD will very soon become a great challenge for public health in Taiwan.

Initially, PD patients exhibit rest tremor, muscular rigidity, gait impairment and postural instability that is not caused by primary visual, vestibular, cerebellar, or proprioceptive dysfunction. Specific non-motor features become obvious as the disease progresses (olfactory dysfunction, cognitive impairment, psychiatric symptoms, sleep disorders, autonomic dysfunction, pain, and fatigue), resulting in clinically significant disability [1]. The shift from impairment to disability in PD occurs within 3 to 7 years after diagnosis [5]. Patients with late-stage disease tend to experience axial motor symptoms such as postural instability with frequent falls and freezing of gait [1]. The process becomes risk of developing aspiration pneumonia. Psychological problems often accompany including anxiety, depression, insomnia, cognitive problems and hallucinations [6]. Long-term complications of dopaminergic therapies for PD are characterized by fluctuations, dyskinesia and psychosis, all of which contribute to disability [1]. In late-stage disease, levodopa-resistant symptoms contribute to disability and markedly increase the risk of all-cause mortality [1].

Clinical evidence suggests that the progression of PD is aggravated by vascular risk factors including diabetes [7] and hypertension [8]. Otherwise PD twice the general population to develop cardiovascular disease and have a 50% greater chance of dying from it [9]. Some researchers have found that PD is linked to an increased risk of stroke and a higher risk of stroke-related mortality [10, 11]. However a number of studies have demonstrated that traditional Chinese medicine (TCM) [12–15], acupuncture [16, 17], and Tai Chi [18] improve impairments associated with neurologic sequelae. Since none of the currently available treatments for PD are specifically designed to prevent stroke, this therapeutic area presents an important unmet need. The purpose of this study was to investigate the prophylactic benefit of TCM in the prevention of stroke among patients with PD.

## Materials and methods

### Data source

Taiwan’s single-payer National Health Insurance (NHI) program delivers universal health coverage to its residents [19]. De-identified claims data from the NHI Research Database provided the study participants’ details on gender, date of birth, clinical visits and hospitalizations, medicines prescribed and their dosages (including Chinese herb products), medical expenditure, as well as the disease diagnosis classified according to International Classification of Diseases, Ninth Revision, Clinical Modification (ICD-9-CM) codes. Our study was approved by the Ethics Review Board of China Medical University (CMUH104-REC2-115).

### Study subjects

**Fig 1:**
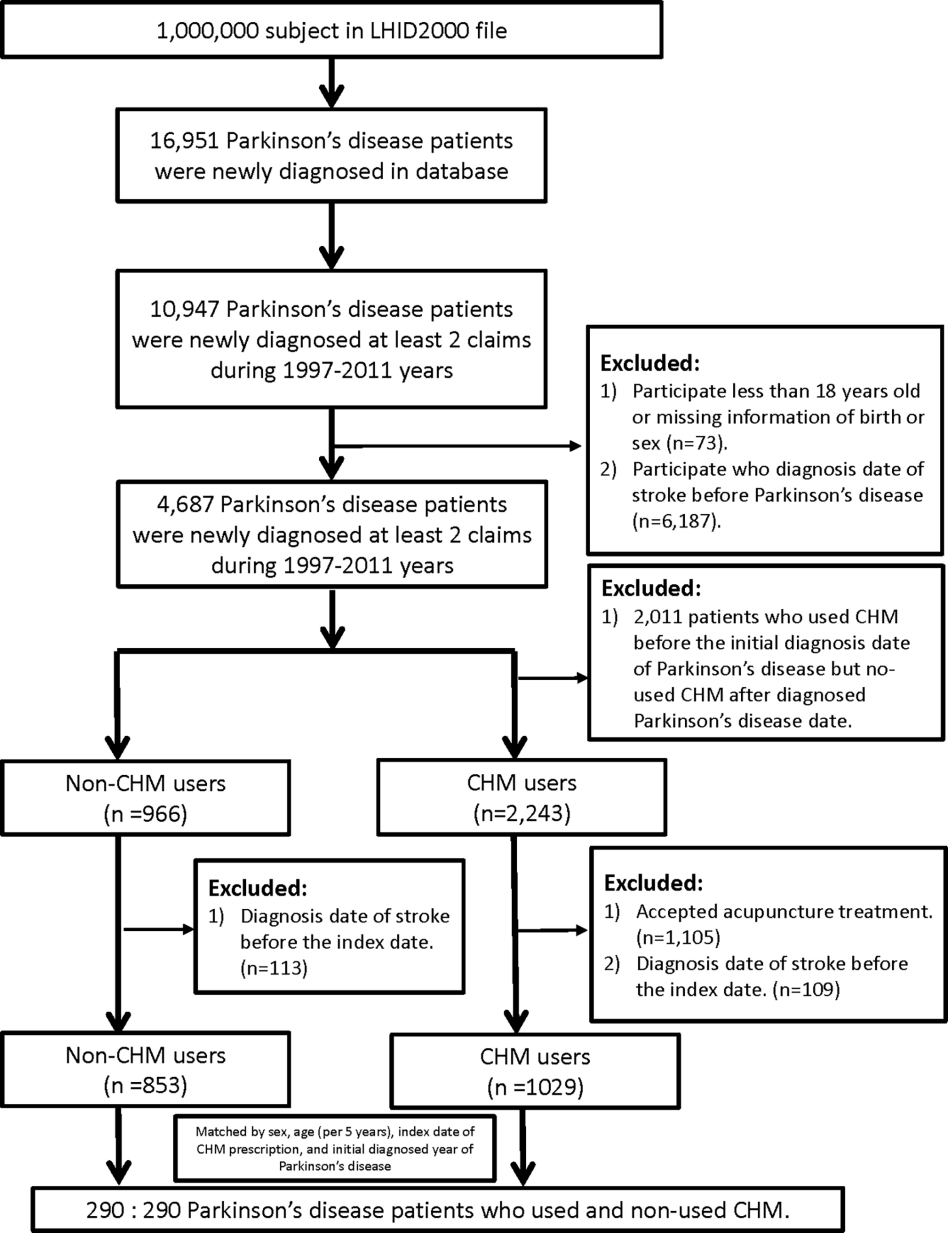
Flowchart for the patient-selection from the Longitudinal Health Insurance Database in Taiwan.

Study subjects were selected from the Longitudinal Health Insurance Database (LHID2010) (Fig 1). The LHID2010 contains registration and claims data of 1,000,000 NHI beneficiaries randomly sampled from all those enrolled between Jan. 1, 2010 and Dec. 31, 2010. Gender distribution does not differ significantly between patients in the LHID2010 and the original National Health Insurance Research Database (NHIRD). We identified all individuals aged older than 18 years with newly-diagnosed PD (ICD-9-CM code 332.x) who made at least two visits seeking clinical medical care with diagnosis to PD in the entire study period from 1997 to 2011. PD patients who had at least one Chinese herbal medicine (CHM) prescription were defined as CHM users, whereas PD patients who had no such details were defined as non-CHM users. We excluded CHM users who received CHM treatment before the diagnosed date of PD and who received acupuncture intervention. The index date was defined as the first date that PD patients started to receive CHM treatment. Immortal time was defined as the period from the initial diagnostic date of PD to the index date. We then randomly selected non-CHM users as the comparison cohort (ratio 1:1), which were matched by age (in 5-year increments), gender, index year of CHM prescription (initial CHM prescription following the new PD diagnosis date), and initial year of PD diagnosis.

### Measurement outcomes, comorbidities, and medication use

The primary outcome was the occurrence of stroke. Patients with stroke were identified using ICD-9-CM codes 430 to 438 with at least 2 ambulatory claims or at least 1 claim for inpatient care. Patients who diagnosed with stroke before the index date of CHM prescription were excluded. All patients were traced from the index date of CHM prescription to the date of stroke diagnosis, withdrawal from the NHI program, or to the end of 2013. The following comorbidities identified before the index date were considered to be potential confounding factors for PD: hypertension (ICD-9-CM code 401 to 405), hyperlipidemia (ICD-9-CM code 272), and diabetes mellitus (DM, ICD-9-CM code 250). The following comorbidities identified before the index date were also considered to be potential confounding factors for stroke: artial fibrillation (AF, ICD-9-CM code 427.31), transient ischemic attack (TIA, ICD-9-CM code 435.9), congestive heart failure (CHF, ICD-9-CM code 428.0), and peripheral vascular disease (PVD, ICD-9-CM code 443.9). Our analysis included the following anti-parkinsonian medications: Pramipexole, Ropinirole, Rotigotine, Trihexyphenidyl, Levodopa, Entacapone, Rasagiline, Aspirin, and Plavix.

### Statistical analysis

Baseline demographic variables and potential risk factors in the CHM group and non-CHM group were analyzed using Chi-Square and t-test calculations for categorical variables and continuous variables, respectively. The crude hazard ratios (HR) and 95% confidence intervals (CI) of stroke in both cohort groups were estimated by time-independent Cox proportional hazard regression models; incidence rates of stroke were calculated after stratifying each variable. Kaplan-Meier analysis of the cumulative incidence of stroke was performed for both groups. All analyses were carried out using SAS statistical software (version 9.4 for Windows; SAS Institute, Inc., Cary, NC, USA). Statistical significance was determined with P <0.05.

## Results

Eligible study participants included 1,882 patients with PD; 853 had used CHM and 1,029 had not. After matching the cohorts, a total of 580 patients were eligible for the study (CHM users: N = 290; non-CHM users: N = 290). The mean age (standard deviation) was 70.64 (±11.58) years in the CHM users and 70.74 (±11.59) years in the non-CHM users. Study subjects were predominantly male (55.17%) and older than 70 years (65.17%). Compared with non-CHM users, a significantly higher proportion of CHM users had hyperlipidemia (CHM user: 34.14%, non-CHM user: 21.03%; P <0.001) and anti-parkinsonian drugs prescription (CHM users: 87.24%, non-CHM user: 73.45; P<0.001). No significant difference has been found in the proportion of stroke-related risk factors between CHM and non-CHM users. Mean follow-up durations were 3.79 years for the CHM users and 2.01 years for the non-CHM users. (Table 1)

**Table 1:** Demographic characteristics of the patients with Parkinson's disease in Taiwan between 1997–2011.

Variable	Without CHM		With CHM		p value
	n = 290(50%)		n = 290(50%)		
	n	%	n	%	
**Sex**					0.99^#^
Female	130	44.83	130	44.83	
Male	160	55.17	160	55.17	
**Age at baseline, years**					0.99^#^
18–59	36	12.41	36	12.41	
60–69	65	22.41	65	22.41	
Older than 70 years	189	65.17	189	65.17	
Mean(SD)	70.74(11.59)		70.64(11.58)		0.9251^‡^
**Baseline comorbidities**					
Hypertension	175	60.34	173	59.66	0.8654^#^
Hyperlipidemia	61	21.03	99	34.14	0.0004^#^
DM	79	27.24	93	32.07	0.2031^#^
AF	7	2.41	5	1.72	0.5596^#^
TIA	0	0	0	0	-
CHF	20	6.9	14	4.83	0.2889^#^
PVD	3	1.03	6	2.07	0.5044†
^§^**Drug used (after initial diagnosis date of Parkinson's disease)**					0.0919^#^
No	77	26.55	37	12.76	
Yes	213	73.45	253	87.24	
**Follow-up period, year (mean, median)**	2.01(0.87)		3.79(2.54)		<0.0001^‡^
**Interval between onset of Parkinson's disease and the first CHM consultation, days mean (median)**			345.48(168.5)		

Abbreviate: CHM, Chinese herb medicine; DM, diabetes mellitus; AF, artial fibrillation; TIA, transient ischemic attack; CHF, congestive heart failure; PVD, peripheral vascular disease; SD, standard deviation.

‡ t test

^#^ Chi-square test

† Fisher exact test.

§Drug included Pramipexole, Ropinirole, Rotigotine, Trihexyphenidyl, Levodopa, Entacapone, Rasagiline, Aspirin, and Plavix.

**Fig 2:**
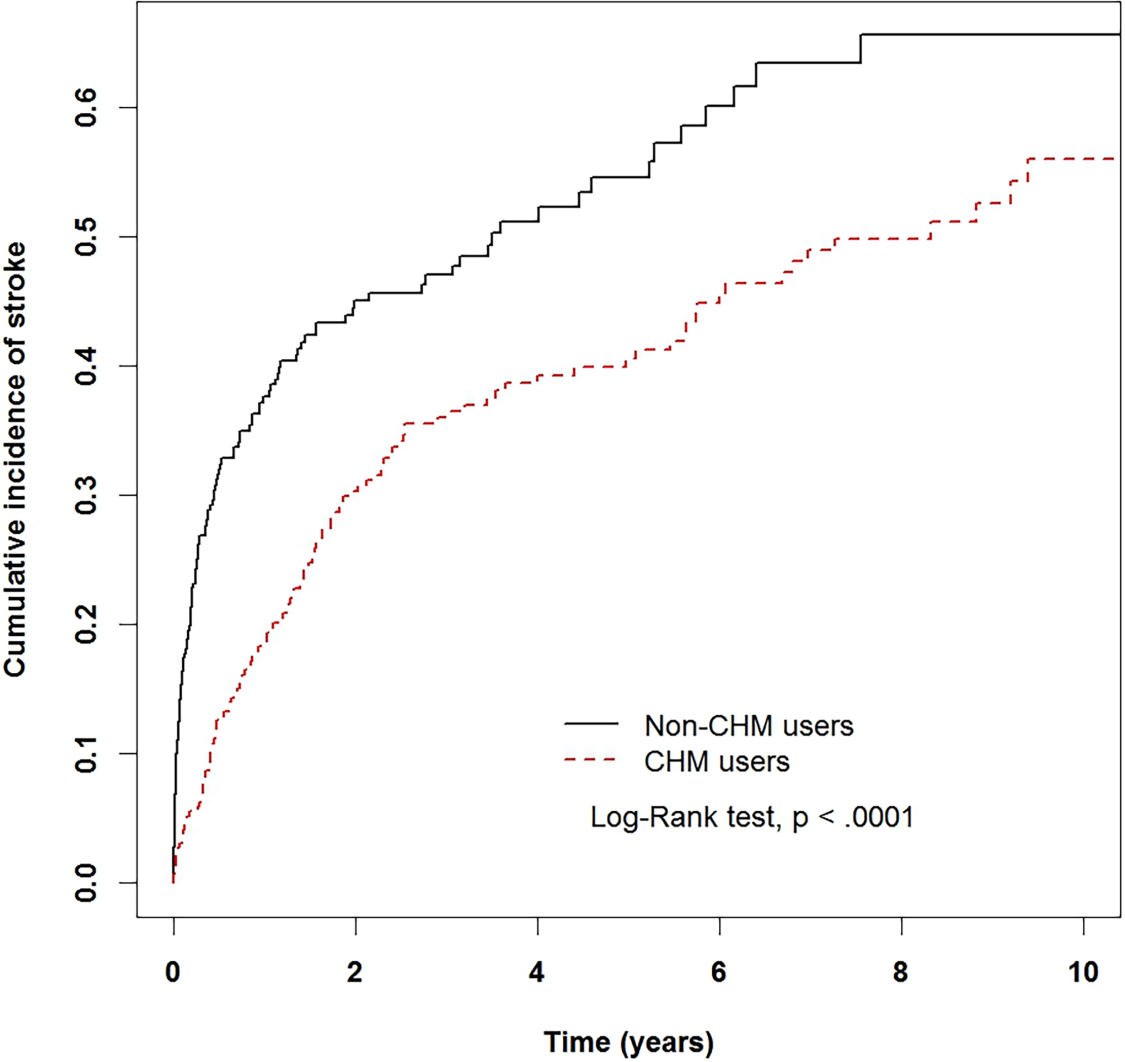
The estimated cumulative incidence of stroke between the Chinese herb medicine (CHM) users and non-CHM users cohort among Parkinson’s disease patients by Kaplan-Meier analysis.

In Kaplan-Meier analysis, the cumulative incidence curve for stroke was significantly lower in the CHM users than in the non-CHM users (log-rank test P <0.001) (Fig 2). PD patients using CHM had a 40% lower risk of stroke compared with PD patients who did not use CHM (unadjusted model, HR: 0.6; 95% CI: 0.47 to 0.77; P <0.001). This beneficial effect was only slightly modified after adjusting for sociodemographic factors and comorbid conditions (adjusted HR [aHR]: 0.59; 95% CI: 0.46 to 0.76; P <0.001) (Table 2). Besides, compared with patients in the 18–59-year age group, those who aged between 60 and 69 years had a higher risk of stroke (aHR: 3.91; 95% CI: 1.92 to 7.95; P <0.001) as did those who aged over 70 years (aHR: 4.76; 95% CI: 2.4 to 9.42; P <0.001). Patients with hypertension also had a higher risk of stroke (aHR: 1.61; 95% CI: 1.21 to 2.15; P = 0.0011). Patients prescribed with anti-parkinsonian drugs had a lower risk of stroke (aHR: 0.61; 95% CI: 0.46 to 0.82; P = 0.0012) (Table 2).

**Table 2:** Cox model measured hazard ratio and 95% confidence intervals of new-onset stroke in Parkinson's disease patients.

Variable	Stroke no. (n = 257)	Crude			Adjusted		
		HR	(95% CI)	P-value	HR	(95% CI)	P-value
**CHM used**							
Non-TCM users	135	1.00	reference		1.00	reference	
TCM users	122	0.60	(0.47–0.77)	<0.0001	0.59	(0.46–0.76)	< .0001
**Sex**							
Female	120	1.00	reference		1.00	reference	
Male	137	0.95	(0.75–1.22)	0.7063	0.98	(0.76–1.26)	0.8529
**Age, years**							
18–59	9	1.00	reference		1.00	reference	
60–69	59	4.42	(2.19–8.91)	<0.0001	3.91	(1.92–7.95)	0.0002
Older than 70 years	189	5.62	(2.88–10.98)	<0.0001	4.76	(2.41–9.42)	< .0001
**Baseline comorbidity**							
Hypertension	181	1.89	(1.45–2.47)	<0.0001	1.61	(1.21–2.15)	0.0011
Hyperlipidemia	72	0.96	(0.73–1.27)	0.7923	0.83	(0.61–1.13)	0.234
DM	77	1.06	(0.81–1.38)	0.693	1.00	(0.75–1.34)	0.9942
AF	5	1.16	(0.48–2.81)	0.7469	0.91	(0.36–2.3)	0.8502
CHF	15	1.17	(0.70–1.98)	0.5465	1	(0.58–1.74)	0.9902
PVD	6	1.51	(0.67–3.40)	0.3175	1.25	(0.55–2.82)	0.5973
**Drug used**^§^							
No	61	1.00	reference		1.00	reference	
Yes	196	0.54	(0.41–0.72)	< .0001	0.61	(0.46–0.82)	0.0012

Adjusted HR: Adjusted for CHM used, age, sex, baseline comorbidity and drug used in Cox proportional hazards regression.

Abbreviation: HR, hazard ratio; CI, confidence interval; CHM, Chinese herb medicine; DM, diabetes mellitus; AF, artial fibrillation; CHF, congestive heart failure; PVD, peripheral vascular disease.

§ Drug included Pramipexole, Ropinirole, Rotigotine, Trihexyphenidyl, Levodopa, Entacapone, Rasagiline, Aspirin, and Plavix.

We then explored the association between CHM prescription and stroke in analyses stratified by age, sex, comorbidities and medication use (Table 3). Overall, the incidence rates of stroke for CHM users and non-CHM users were 11.10 per 100 person-years and 23.15 per 100 person-years, respectively. Compared with non-CHM user, using CHM was associated with a significantly lower risk of stroke among both females (aHR: 0.62; 95% CI, 00.42 to 0.91; P <0.05) and males (aHR: 0.54; 95% CI: 0.38 to 0.77, P <0.001); patients aged over 70 years (aHR 00.56; 95% CI, 0.42 to 0.76; P <0.001); patients with hypertension (aHR: 0.53; 95% CI: 0.39 to 0.72; P <0.001); patients without hyperlipidemia (aHR: 0.519; 95% CI: 0.37 to 0.68; P <0.001), DM (aHR: 0.57; 95% CI: 0.42 to 0.77; P <0.001), AF (aHR: 0.60; 95% CI: 0.46 to 0.77; P <0.001), CHF (aHR: 0.56; 95% CI: 0.43 to 0.72; P <0.001), and PVD (aHR: 0.58; 95% CI: 0.45 to 0.75; P <0.001); and in those who did not use PD-related medications (aHR: 0.52; 95% CI: 0.29 to0.93; P <0.01) as well as those who did (aHR: 0.58; 95% CI: 0.43 to 0.78; P <0.001). However, using CHM was associated with significantly higher risk of stroke than non-CHM user when patient have CHF (aHR: 16.2; 95% CI: 2.11 to 124.33, P <0.01).

**Table 3:** Incidence rates, hazard ratio and confidence intervals of disorders of stroke association with and without CHM usage among Parkinson's disease patients in the stratification of sex, age, comorbidity and drug used.

Variables	CHM used						Compared with non-CHM users	
	No			Yes			Crude HR	Adjusted HR
	(n = 290)			(n = 290)				
	Event	Person years	IR	Event	Person years	IR	(95%CI)	(95%CI)
**Total**	135	583	231.54	122	1099	111.01	0.60(0.47–0.77)***	0.59(0.46–0.76)***
**Sex**								
Female	60	290	206.75	60	496	121.01	0.69(0.48–0.99)*	0.62(0.42–0.91)*
Male	75	293	256.11	62	603	102.79	0.52(0.37–0.74)***	0.54(0.38–0.77)***
**Age, years**								
18–59	6	138	43.41	3	169	17.76	0.44(0.11–1.76)	0.69(0.15–3.13)
60–69	30	155	193.74	29	269	107.97	0.68(0.41–1.15)	0.65(0.38–1.13)
Older than 70	99	290	341.39	90	662	136.05	0.53(0.40–0.71)***	0.56(0.42–0.76)***
**Baseline comorbidity**								
Hypertension								
No	37	290	127.42	39	491	79.49	0.75(0.48–1.18)	0.76(0.48–1.23)
Yes	98	293	334.85	83	608	136.42	0.53(0.39–0.71)***	0.53(0.39–0.72)***
Hyperlipidemia								
No	109	438	248.93	76	759	100.16	0.53(0.39–0.71)***	0.51(0.37–0.68)***
Yes	26	145	179.09	46	340	135.21	0.8(0.49–1.29)	0.92(0.54–1.57)
DM								
No	99	455	217.42	81	749	108.19	0.60(0.45–0.81)***	0.57(0.42–0.77)***
Yes	36	128	281.87	41	350	117.04	0.59(0.37–0.93)*	0.63(0.39–1.01)
AF								
No	131	575	227.67	121	1082	111.84	0.61(0.48–0.78)***	0.6(0.46–0.77)***
Yes	4	8	521.79	1	17	58.33	0.23(0.03–2.14)	-
CHF								
No	129	544	236.96	113	1068	105.84	0.56(0.44–0.73)***	0.56(0.43–0.72)***
Yes	6	39	155.19	9	31	287.15	1.87(0.66–5.26)	16.2(2.11–124.33)**
PVD								
No	133	576	230.78	118	1086	108.67	0.59(0.46–0.76)***	0.58(0.45–0.75)***
Yes	2	7	296.23	4	13	304	1.12(0.12–10.33)	-
**Drug used**^§^								
No	48	119	404.84	34	259	131.29	0.50(0.32–0.79)**	0.51(0.33–0.81)**
Yes	87	464	187.3	88	840	104.76	0.65(0.48–0.88)**	0.59(0.44–0.80)***

Adjusted HR: adjusted for CHM used, age, sex, baseline comorbidity and drug used in Cox proportional hazards regression.

Reference group was non-CHM users.

Abbreviation: IR, incidence rates, per 1,000 person-years; HR, hazard ratio; CI, confidence interval; CHM, Chinese herb medicine; DM, diabetes mellitus.

§ Drug included Pramipexole, Ropinirole, Rotigotine, Trihexyphenidyl, Levodopa, Entacapone, Rasagiline, Aspirin, and Plavix.

*:< 0.05

**:< 0.01

*** p<0.001

Table 4 compares medical expenditures for hospitalization and outpatient care between CHM users and non-CHM users over the first 3 months, 3 years and 5 years. Except mean medical expenditure of outpatients care was higher in over five years, in all three periods the medical expenditure was lower (but not significantly) among CHM users compared with non-CHM users.

**Table 4:** Medical expenditure of hospitalization and outpatient care between CHM and non-CHM users for the first six months, 3years and 5years after incident Parkinson's disease.

Cost	Non-CHM users		CHM users		P-value^§^
	n	mean(SD)	n	mean(SD)	
**First 3 month**					
Outpatients care, NT	283	17159(24955)	289	14372(15246)	0.1085
Hospitalization, NT	74	149143(363738)	46	69764(136141)	0.093
**First 3 years**					
Outpatients care, NT	288	132047(201875)	290	130763(95079)	0.9222
Hospitalization, NT	193	284805(560630)	160	215760(337874)	0.1546
**First 5 years**					
Outpatients care, NT	288	182836(283977)	290	191529(138882)	0.6407
Hospitalization, NT	213	353634(681285)	194	258495(405785)	0.0847

Abbreviation: CHM, Chinese herb medicine; SD, standard deviation.

§ t-test.

Finally, we analyzed prescription patterns of Chinese herbs issued by TCM doctors. Many CHM in single herbal preparations or in formulas of multiple herbs have been prescribed for CHM users. The two most commonly used single herbal preparations consisted of Danshen and Rhubarb; the two most common herbal formulas were Ma Zi Ren Wan and Du Huo Ji Sheng Tang. The average prescription duration of ten most commonly used single herbal preparations and herbal formulas were 7.53 days and 8.12 days, respectively (Table 5). There is no significant correlation between the daily CHM dosage and the risk of stroke (Table 6).

**Table 5:** Ten most common single herbs and herbal formulas prescribed.

	Frequency	Number of person-days	Average daily dose (g)	Average duration for prescription (days)
**Single herb**				
Danshen	188	1363	6.1	7.3
Rhubarb	159	1242	0.8	7.8
Gouteng	150	1459	1.3	9.7
Huangqi	105	768	1.4	7.3
Ophiopogon	103	725	2.6	7
Millettia	102	828	4.2	8.1
Magnolia	102	711	1	7
Lotus seeds	92	638	2.6	6.9
Licorice	90	615	1.2	6.8
Danggui	89	659	1.3	7.4
**Herb formula**				
Ma Zi Ren Wan	258	2849	7	11
Du Huo Ji Sheng Tang.	215	1591	8.6	7.4
Shujing Huoxue Tang	162	1231	4.5	7.6
Ganmai jujube soup	145	1078	4	7.4
Suanzaoren soup	131	981	5.8	7.5
Liu Wei Di Huang Wan	117	799	4	6.8
Jia Weixiao yao san	117	1065	3.7	9.1
Tian Wang Bu Xin Dan	108	996	4	9.2
Shao Yao Gan Cao Tang	103	827	4	8
Bu Yang Huan Wu Tang	93	665	10	7.2

**Table 6:** Hazard Ratios and confidence intervals of stroke risk associated with average dosage per day of CMH.

Dosage of CMH/per day	N	No. of event	Crude HR	Adjusted HR
			(95% CI)	(95% CI)
**Chinese herb users**				
Less than 12.68 (g)	72	32	1(reference)	1(reference)
12.68–14.23 (g)	71	32	0.99(0.61–1.62)	1.06(0.64–1.75)
More than 14.23 (g)	147	58	0.97(0.63–1.50)	1.06(0.68–1.66)

Adjusted HR: adjusted for CHM used, age, sex, baseline comorbidity and drug used in Cox proportional hazards regression.

Abbreviation: HR, hazard ratio; CI, confidence interval; CHM, Chinese herb medicine; DM, diabetes mellitus.

## Discussion

In the present study, we evaluated whether CHM treatment reduced the risk of stroke among PD patients using a Longitudinal Health Insurance Database in Taiwan. Of all 1,882 PD patients who were included in this study, almost half (N = 853; 45%) had ever took CHM. After controlling several potential confounding factors, the CHM users among patients with PD revealed a significant decrease in risk of new-onset stroke than matched non-CHM users, expect the patients with CHF. Among these CHM users, the most commonly used single herb is Danshen and the most common herbal formula is Ma Zi Ren Wan.

In our multivariable Cox proportional hazard analysis, the risk of stroke after PD diagnosis was significantly reduced among CHM users compared with non-CHM users. After adjusted for residual confounding effects, the CHM users still have significant lower risk than non-CHM users even in the subgroup with higher risk of stroke (i.e. PD patient who aged over 70 years and who with hypertension). The reduction of the risk may be benefit by the prevention effect of the CHM on cardiovascular disease. Although the underlying mechanisms are not fully explored, recent studies suggest that the prevention and treatment role of CHM on cardiovascular diseases may achieved via the mechanism of antioxidants, anti-inflammation, and anti-apoptosis [20]. Our analysis on the prescription patterns of CHM further support the notion of the prevention effect of the CHM on cardiovascular disease. Danshen, the dried root of *Salvia miltiorrhiza*, is used to promote blood flow and treat vascular disease [21]. Its pharmacologic mode of action may help to prevent the development of atherosclerosis and it may also have antihypertensive and anti-platelet aggregation effects, helping to prevent cerebral infarction [22]. The formula Du Huo Ji Sheng Tang effectively attenuates inflammation and promotes lymphatic drainage in the TNF-α-transgenic mouse model of rheumatoid arthritis (RA) [13]. Du Huo Ji Sheng Tang has long been used in China to treat patients with osteoarthritis and is well-recognized for its ability to improve patients’ clinical symptoms and knee function [23]. Hence, the prevention effect of the CHM on new-onset stroke in PD patients may be achieved through the mechanisms of antioxidants, anti-inflammation, and anti-apoptosis.

The alternative explanation for the prevention effect of the CHM might be the life style of the PD patients. A nationwide survey conducted in Taiwan during 2002 and 2003 revealed that people who exercised regularly were more likely to use TCM services than people who did not exercise regularly [24]. In comparison to patients with PD who do choose not to use TCM, those who do may have more positive attitudes towards physical rehabilitation and disease prevention, which may contribute to the reduction of new-onset stroke events after PD diagnosis. However, the same survey also revealed that people with self-reported poor health status are more likely to use TCM services than people who consider they have very good health status [24], indicating that TCM users have more comorbidities than their healthier counterparts and therefore require more healthcare. In the present study, a significantly higher proportion of CHM users compared with non-CHM users had hyperlipidemia, which is known to increase the risk of stroke secondary to atherosclerosis [25], indicating a poor health state in CHM users. Although there appeared to be no such association for the occurrence of stroke in our study, there is no significant difference between CHM and non-CHM users on the risk of stroke in the PD patients with hyperlipidemia. Thus, the life style might be a minor factor to reduce the risk of ischemic stroke on PD patients.

An interesting finding in the present study is that the Ma Zi Ren Wan formula has been most common prescription of CHM on PD patients. The Ma Zi Ren Wan formula has been used for over two thousand years in China and other Asian countries to treat constipation [26]. A recent systematic review and meta-analysis of 9 studies involving a total of 741,593 participants reported that people with constipation were more than twice as likely (pooled odds ratio, 2.27; 95% CI, 2.09 to 2.46) to develop subsequent PD than those without constipation [27]. Thus, it raised a possibility that CHM may also have the prevention or treatment effect in PD [28]. Previous studies demonstrated that Chinese herb and herbal extracts have neuroprotective effect on dopaminergic neurons by the distinct mechanisms as antioxidants, free radical scavengers, and anti-apoptosis activity [29]. A recent, large, population-based follow-up study has reported a significantly increased risk of ischemic stroke among patients with PD compared with propensity score-matched subjects without PD, whereas the increased risk of ischemic stroke in PD patients may associated with the common risk factors between PD and ischemic stroke, e.g. oxidative stress, diabetes, and hypertension [11]. Hence, it is plausible that the mechanisms which underlying the prevention effect of CHM on stroke in PD patients may also contribute to the prevention or treatment effect of CHM in PD. However, there is no detailed patient information (e.g., lifestyles, physical, mental and laboratory examinations) in the LHID2010 database. Further randomized clinical trials are needed to clarify whether these CHM prescription help to prevent PD.

Notably, although there were too few samples for statistical significance, our study found that patients with PD who received CHM treatment were less costly to care for, whether they were outpatients or hospitalized, during the first 3 months to first 3 years after their PD diagnosis. Moreover, the cost of hospitalization care still less in CHM users than non-CHM users even 5 years after PD has been diagnosed. Since the hospitalization care mostly has been conducted for serious medical conditions, the lower cost of hospitalization care in CHM users may partly contributed by the lower risk of stroke in PD patient. On the other hand, the cost of outpatient care was less reduced than hospitalized care in CHM user, and even slightly higher in 5 years after PD has been diagnosed. These results may partly due to the combined cost from both outpatient department of TCM and western medicine, which may conceal the true reduction effect on medical expenditure from CHM prescription. Our findings are echoed by other Taiwanese researches reporting that TCM is associated with the lowest total expenditure per person-time when compared with Western medicine and integrated Chinese-Western medicine among people seeking treatment for allergic rhinitis [30] and that acupuncture treatment reduces use of emergency care and hospitalization in patients with traumatic brain injury [31].

The advantages of this study include the large sample size and use of claims data from the LHID2010, containing 1,000,000 random samples from all beneficiaries in the NHIRD in 2010. Furthermore, time-independent multivariate Cox proportional hazards analysis controlled for residual confounding bias. Study limitations include its use of retrospective medical claims data, which lack detailed patient information about lifestyles, physical, mental and laboratory examinations. Moreover, we used the ICD-9-CM code for PD, without any evidence as to the level of disease severity. As mentioned in the materials and methods, it is also very important to point out that the immortal time bias may exist in this cohort study because of the use of a time fixed analysis. Future prospective studies should include these variables to conquer these limitations.

## Conclusions

The results of this nationwide retrospective cohort study suggest that PD patients using CHM treatment are at lower risk of new-onset stroke compared with PD patients who do not use CHM treatment. The association between CHM treatment and decreased risk of stroke among PD patients existed for both females and males, and for all age groups. The use of CHM by patients with PD in Taiwan is common. Additional pharmacologic investigations or clinical trials could be conducted to further validate the efficacy of commonly prescribed CHM. Overall, our study provides valuable information and adds value to existing knowledge about healthcare for patients with PD, and may provide directions for the design of future therapeutic strategies.
